# Effects of voluntary exercise on apoptosis and cortisol after chronic restraint stress in mice

**DOI:** 10.20463/jenb.2016.09.20.3.3

**Published:** 2016-09-30

**Authors:** Hyobin Seo, Chun-Hyung Park, Seokrip Choi, Woocheol Kim, Byung-Duk Jeon, Seungpil Ryu

**Affiliations:** 1 Department of Leisure Sports, Kyungpook National University, Sangju Republic of Korea; 2 Department of Sports Rehabilitation, Daegu Health College, Daegu Republic of Korea; 3 Department of Physical Education Leisure, Suseong College, Daegu Republic of Korea

**Keywords:** Restraint stress, Voluntary exercise, Apoptosis, Cortisol, Mice

## Abstract

**[Purpose]:**

To determine whether voluntary exercise (wheel running) has the potential of relieving stress.

**[Methods]:**

In this study, restraint stress with or without voluntary wheel running was performed for mice housed in individual cages. A total of 21 ICR male mice were assigned into control (CON), restraint stress with voluntary exercise (RSVE), or restraint stress (RS) without voluntary exercise groups (n = 7 each).

**[Results]:**

No significant difference in body weight increase was found among the three groups, although CON and RS groups had a tendency of having smaller body weight increase compared to the RSVE group. No significant difference in the expression level of liver heat shock protein 70, Bcl-2, or p53 was found among the three groups. However, caspase-3 protein level in RS group was significantly higher than that in the other two groups. Blood cortisol concentration in RS was higher (p < 0.05) than that in RSVE or CON group. It was the lowest (p < 0.05) in the RSVE group.

**[Conclusion]:**

Our findings suggest that apoptosis caused by chronic restraint stress might be suppressed by voluntary exercise in mice.

## INTRODUCTION 

It has been reported that repeated and stressful situation can cause chronic stress in animals and lead to hypothalamus-pituitary-adrenal axis change^1^. In addition, short-term stress can be used as a starting point to determine various hormonal changes in animals^2^. Restraint stress can reduce serum levels of Bcl-2, estradiol, and IGF-I but increase the levels of cortisol and progesterone^3^. From another perspective, carbohydrates and saturated fatty acids have been reported to be preferred by people with chronic stress, resulting in weight gain^4^. It has been reported that the concentration of cortisol in Cynomolgus monkey is increased by restraint stress^5^. On the other hand, exercise can enhance resistance to stress and suppressed anxiety. It can also reduce depression in human and animals^6^. After prolonged repetitive restraint stress, female rats have shown higher concentration of ovarian hormone after treadmill running exercise training^7^. In addition, voluntary exercise of the extremities in stroke patients can assist in the recovery of damaged limbs and their function^8^. Restraint stress is greater than the stress caused by forced swimming exercise^2^. Heat shock protein 72 (HSP 70), a representative marker of stress protein, can be increased by exercise^9^. Exercise can also increases the expression levels of superoxide dismutase of Mn isoform (Mn-SOD), Bax, and caspase-3 protein that inhibit apoptosis in aged rat heart^10^. In addition, 60 min of exercise training can increase the level of Bcl-2 while decreasing the levels of caspases 9, 3, 8, and 12^11^. However, high-intensity exercise can induce cell death and leukocytic apoptosis^12^^,^^13^. 

Chronic stress can suppress elements of the hypothalamus-pituitary-adrenal cortex axis and the biosynthesis of melanin in the skin ^14^. Although the activity of hypothalamus-pituitary-thyroid axis is suppressed by restraint stress, light stress and dietary restrictions are not related to voluntary exercise^15^. Hippocampus neurons are noticeably increased by voluntary exercise, which could prevent damage to memory associated with chronic stress^16^. In addition, inhibition of antibacterial activities and gut function induced by chronic restraint stress can be suppressed by light activities^17^. 

However, forced exercise after restraint stress does not have beneficial effect for impaired memory in rats^18^. Similarly, involuntary exercise can increase the anxiety levels and decrease the levels of vascular endothelial growth factor and brain derived neurotropic factor, both of which function in neuro-biosynthesis in the hippocampus^19^. On the other hand, moderate-intensity treadmill exercise or voluntary exercise can inhibit apoptosis^20^. It has been reported that apoptosis is suppressed and short-term memory impairment is inhibited in diabetic rats by treadmill exercise for 4 weeks after birth^21^. Cortisol concentration is also increased with both forced swim exercise and restraint stress^2^. Similarly, voluntary exercise, not forced exercise, after withdrawal symptoms of drug addiction can relieve stress^22^. The most common method used to train mouse is treadmill running with electrical shock. However, the electrical shock increases stress hormones and cytokines after the exercise^23^. Compared to forced exercise, voluntary exercise appears to provide more reliable results. Therefore, we examined the changes of apoptosis-related protein expression level and cortisol concentration with voluntary exercise after restraint stress in this study. 

## METHODS 

### Subjects 

In this study, 21 male ICR (Institute of Cancer Research) mice at 5 weeks of age were used as subjects. Mice were housed in individual cages with temperature of 23-25℃, relative humidity of around 60%, and a 12-hour interval of light-dark cycle. They were assigned to the following three groups: control (CON), restraint Stress (RES), and restraint stress + voluntary wheel running (RSVE) (n = 7 per group). 

### Dietary composition and food intake 

Commercial feed (formula-m07, Cheil Feed, Korea) was used as diet. Tab water and diet were provided to mice ad libitum. 

### Restraint stress 

**Figure 1.**
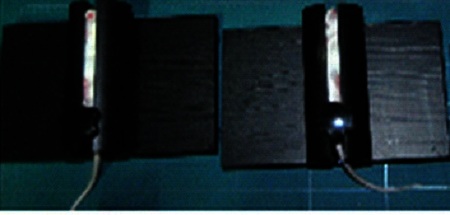
Restraint stress model.

To induce stress, restraint stress is a better method than forced swim exercise^2^. As shown in Figure 1, mice were immobilized for 30 min/d for four weeks^24^. 

### Voluntary wheel exercise 

Voluntary exercise was started when they were 6 weeks old. They ran in a wheel with diameter of 370 mm voluntarily for 4 weeks. They could enter the wheel and go back to their cages freely through a hole between the wheel and cage. In addition, the amount of rotation was counted automatically with a sensor. 

### Sampling 

After 12 hours of fasting, rats were anesthetized with ether. The abdomen was cut open to collect blood samples from the abdominal aorta to analyze blood cortisol concentration. The collected blood samples were centrifuged (1580MER, Gyrozen, Korea) at 700 × g for 10 min. Supernatants were collected and stored in a freezer (NF-400SF, HFC, Japan) until analysis. The liver was removed and fresh frozen in liquid nitrogen to terminate its activation. It was then stored in a -80℃ freezer (NF-400SF, HFC, Japan) until further analysis. 

### Analysis items and Methods

#### Body weight measurement

During the experiment, body weight was measured under the same environment. It was measured every Monday and Thursday at 9:00 AM (twice a week). 

#### Western blot analysis 

To determine protein levels, liver tissue was homogenized in 0.5 M EDTA (Duksan, Korea), lysis buffer (Gendepot, R4200-100, USA), and phosphatase inhibitor 100 x (Gendepot, P3200-001, USA). After homogenization, each sample was centrifuged at 1,200 x g for 10 minutes. Supernatants were collected and subjected to protein concentration measurement using Bradford protein assay method. Briefly, standard BSA or supernatant (1 ㎕) was mixed with 1 ml of Bradford reagent. A 200 ㎕ of the mix was then placed in an ELISA plate to measure the absorbance value at wavelength of 595 nm. After protein concentration measurement, proteins were mixed with Laemmli sample buffer and boiled for 5 min. Protein samples (20μl each) were then loaded into 10% SDS-PAGE gel and run at 100 V to separate the proteins. After electrophoresis, proteins were transferred onto polyvinylidene difluoride (PVDF) membranes by electro-blotting at 100 V at room temperature for 1 h (± 20~30 min). The membranes were incubated with 5% skim milk blocking buffer (Skim milk block buffer; TBST+5% skim milk) for 1 h followed by incubation with primary antibodies at 4℃ overnight. Primary antibodies were prepared by diluting in 5% skim milk. The following primary antibodies were used: HSP70 1:2,000 (Abcam, UK), Bcl-2 1:200 (Santa cruz, USA), p53 1: 1,000 (Abcam, UK), and caspase-3 1:1,000 (Cell signaling, USA). After washing 5 times (10 min each) with TBST buffer (50 mM Tris-Hcl, 150 mM NaCl and 0.05%, Tween 20), membranes were incubated with respective secondary antibodies at room temperature for 1 h. For HSP70 1:5,000, goat-anti rabbit secondary antibody (Santa Cruz, USA) was used. For Bcl-2 1:2,000, goat-anti rabbit secondary antibody (Santa Cruz, USA) was used. For p53 1:5,000, goat-anti mouse secondary antibody (Santa Cruz, USA) was used. For caspase-3 1:3,000, goat-anti rabbit secondary antibody (Santa Cruz, USA). After rinsing with TBST (5% tween-20) 5 times (10 min each), the membranes were incubated with ECL reagent and developed onto X-ray film. Band intensities were measured with Image J (NIH, Ver. 1.47t, USA). Their intensities were normalized to the level of β-actin as loading control. 

#### Cortisol analysis 

Blood cortisol concentrations were measured using an ELISA kit (MyBioSource, CANADA). Briefly, blood samples were centrifuged at 1,000 rpm for 10 minutes and left at room temperature for 20 minutes after diluting the sample with reagent provided by the kit in accordance with the manufacturer’s instructions. Both standard and sample dilutions were performed in 96-well plates. They were incubated at 37℃ after mixing 100 ㎕ for 90 minutes in a shaker. Washing was then performed with buffer three times, followed by the addition of 400 ㎕ to each well after mixing. The plate was turned upside down to remove the wash buffer. Then 100 ㎕ of prepared antibody was added to each well and incubated at 37℃ with shaking for 60 minutes. The plate was then washed with washing buffer three times. Cortisol enzyme-conjugate mixed with buffer (100 ㎕) was then added to each well followed by incubation at 37℃ for 30 minutes with shaking. Finally, washing was performed using 400 ㎕ of washing buffer five times. The plate was turned upside down to remove the wash buffer. After adding 100 ㎕ of a dark color reagent, the 96-well plate was placed on a shaker for mixing, followed by addition of 100 ㎕ of color reagent C. After 30 minutes of mixing, the absorbance value was measured at wavelength of 450 nm on an ELISA reader (Tecan Infinite, F50, Austria). 

### Statistics 

All data analysis in this study were performed with SPSS/PC+20.0, a statistical program designed for Window-based PCs. All test data were presented in averages with standard errors. One-way analysis of variance (ANOVA) was conducted to verify significant differences among groups. In case of an item with significant difference, post verification was carried out using the Least Significant Different method. All significance levels were set at p < 0.05. 

## RESULTS 

### Body weight gain 

**Figure 2.**
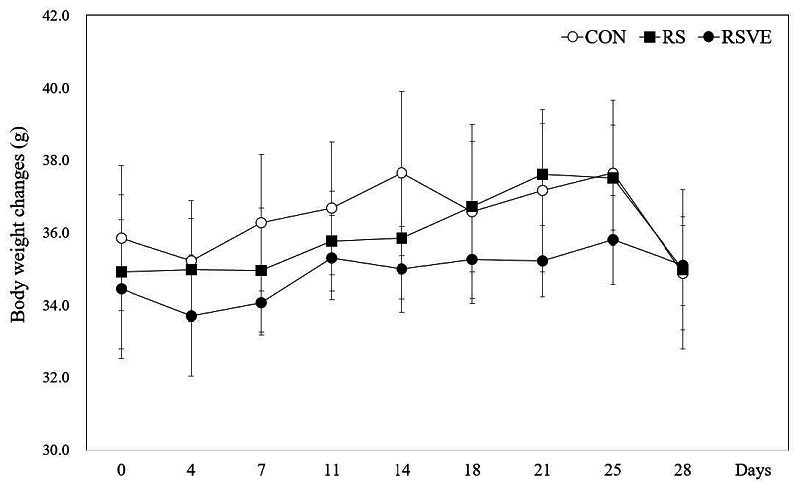
Changes in body weight during the experimental period. Bars are mean ± standard error. CON: control group; RS: restraint stress group; RSVE: restraint stress and voluntary exercise group.

Results of body weight gain are shown in Figure 2. As shown in Figure 2, there was no significant difference in body weight gain among the three groups. However, the body weight gains in CON and RS tended to be smaller compared to that of RSVE group.

### Protein expression level 

**Figure 3.**
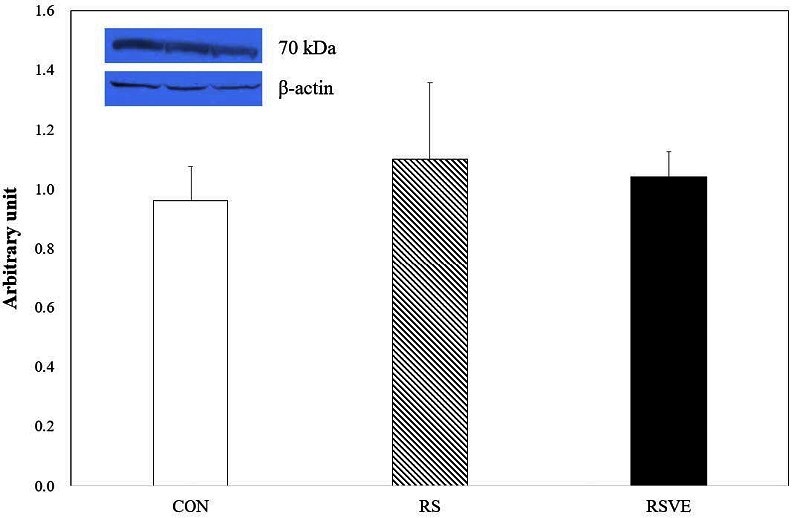
Difference in heat shock protein 70 (HSP70) protein expression. Bars are mean ± standard error. CON and white bar: control group; RS and striped pattern bar: restraint stress group; RSVE and black bar: restraint stress and voluntary exercise group.

**Figure 4.**
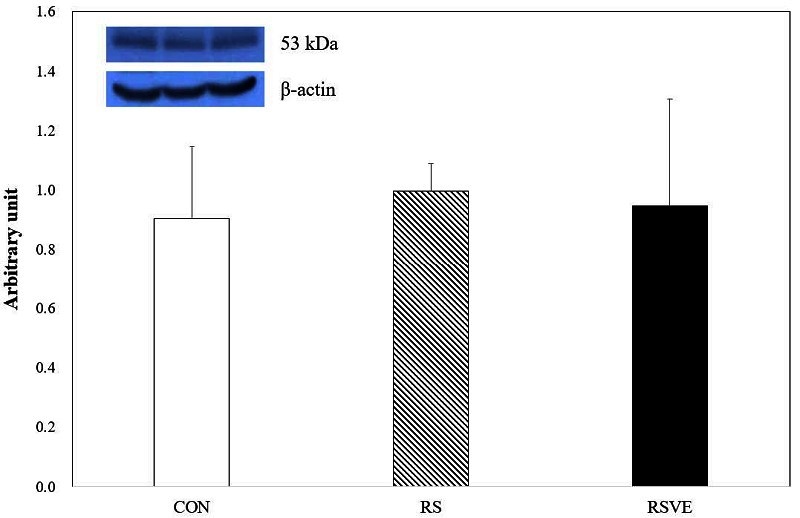
Difference in p53 protein expression. Bars are mean ± standard error. CON and white bar: control group; RS and striped pattern bar: restraint stress group; RSVE and black bar: restraint stress and voluntary exercise group.

**Figure 5.**
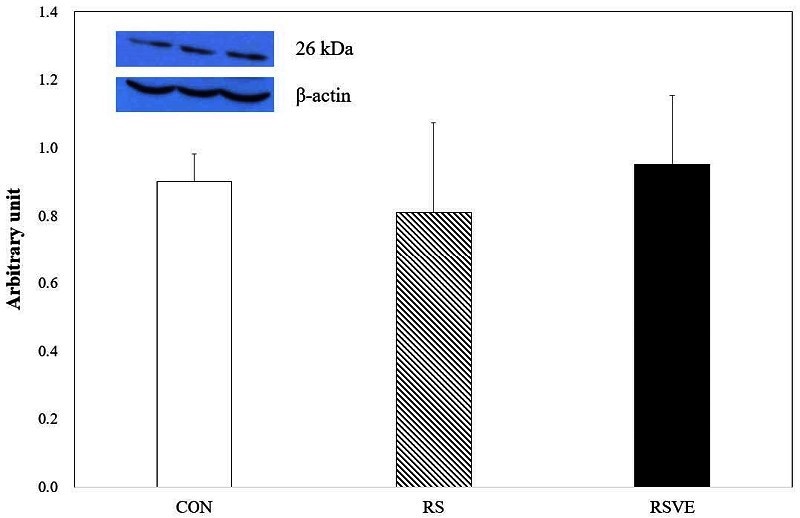
Difference in Bcl-2 protein expression. Bars are mean ± standard error. CON and white bar: control group; RS and striped pattern bar: restraint stress group; RSVE and black bar: restraint stress and voluntary exercise group.

**Figure 6.**
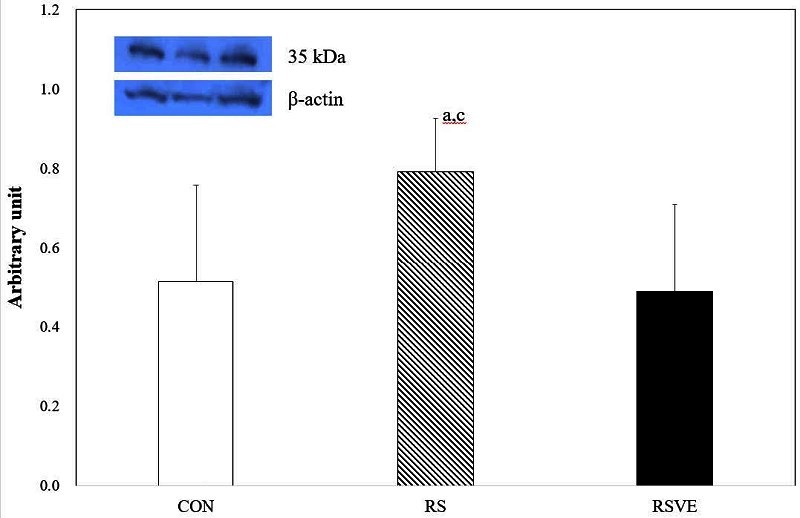
Difference in caspase-3 protein expression. Bars are mean ± standard error. CON and white bar: control group; RS and striped pattern bar: restraint stress group; RSVE and black bar: restraint stress and voluntary exercise group. a: significantly different from CON, p < 0.05; c: significantly different from RSVE, p < 0.05.

Apoptosis-related protein expression levels of HSP70 (Figure 3), p53 (Figure 4), and Bcl-2 (Figure 5) were not statistically different among the three groups. However, caspase-3 protein expression (Figure 6) was significantly (p < 0.05) higher in RS group compared to that in the other two groups.

### Blood cortisol level 

**Figure 7.**
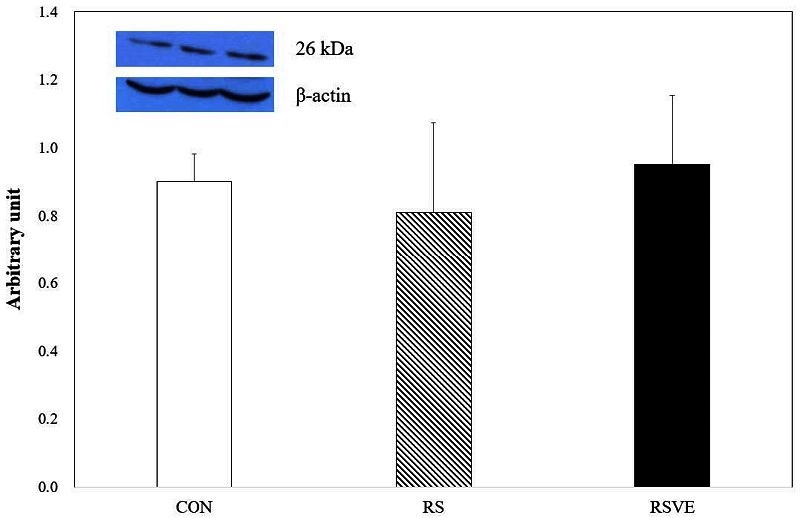
Difference in blood cortisol concentration. Bars are mean ± standard error. CON and white bar: control group; RS and striped pattern bar: restraint stress group; RSVE and black bar: restraint stress and voluntary exercise group. a: significantly different from CON, p < 0.05; b: significantly different form RS, p < 0.05; c: significantly different from RSVE, p < 0.05.

Results of blood cortisol concentrations are shown in Figure 7. The highest concentration of blood cortisol was found in the RS group. The lowest blood cortisol level was found in the RSVE group with statistical significance (p < 0.05). Blood cortisol level in the RSVE was also significantly (p < 0.05) lower than that in CON. 

## DISCUSSION 

In this study, we examined the effect of voluntary exercise on apoptosis and blood cortisol level using chronic restraint stress mice model. It has been reported that relatively gentle exercise for C57BL/6 mice without electrical stimulation during treadmill running with voluntary walking speed may prevent contamination of experimental data^23^. In addition, rats with forced-treadmill exercise have shown more complicated theta wave in the brain and heart rate than rats with voluntary exercise rats^25^. Therefore, we used a voluntary exercise device in this study. 

In general, chronic stress can cause weight loss, especially when restraint stress is loaded. Chronic stress also inhibits weight gain during mice breeding^26^. After restraint stress for 14 days, body weight gain is suppressed from the third day of the experiment. Then a few amount of body weight is recovered after the recovery periods^27^. However, in the present study, there was no difference in weight gain in RS. However, RSVE showed approximately 5% of weight gain with increased amount of skeletal muscles with exercise training. It has been reported that voluntary running exercise can increase the amount of lower limbs’ skeletal muscle^28^^, ^^29^. These results implies that participation of voluntary exercise in a stressful situation that may prevent weight loss. In addition, voluntary exercise can serve as a controller for someone who suffers from many kinds of stress that can cause weight loss despite of the same amount of meal intake^30^. 

Restraint stress can inhibit the biosynthesis of melanin in the skin^13^, weaken the hippocampus and cortical GABAergic neurotransmitters^31^, and induce depression in pregnant rats^32^. It has been reported that heat shock protein 70 (HSP70) is increased under stress situations^33^. Under lipid peroxidation condition, HSP70 level is increased dramatically in a short period of time^34^. In this study, there was no difference in the expression levels of HSP70 among the three groups, although its level was increased by RS. HSP70 can suppress stress-induced myocardial apoptosis through interaction with FAF-1^35^ and stress protein marker HSP 72, resulted in apoptosis suppression in exercised rats^9^. Our results suggest that chronic restraint stress can trigger stress, the lead cause of apoptosis. However, there was no difference among the three groups. Therefore, we carefully analyzed the expression levels of apoptosis-related proteins. 

Meanwhile, p53 protein is a well-known tumor suppressor. Bcl-2 protein expression is increased when the expression of p53 protein is suppressed^36^. Higgins et al.^37^ have reported that exercise can increase p53 protein expression in lung cancer tumor tissue and induce apoptosis in lung cancer. However, p53 protein expression is suppressed even with exercise training^38^. In addition, in stress-induced mice, despite receiving various kinds of vitamins without considering exercise training, only a tendency of p53 reduction is observed whereas the phosphorylation of p53 is increased by exercise^39^. However, there was no significant difference in the levels of p53 among the three groups in the present study, although there was a tendency of a higher expression of p53 in RS and lower expression in RSVE. 

Anti-apoptosis bcl-2 protein can refrain from apoptosis. When morphine-dependent apoptosis in induced, voluntary exercise or exercise participation can increase the level of bcl-2 protein expression, thus suppresses apoptosis^20^. However, its expression is not increased by acute high-intensity swimming exercise^12^. Various kinds of stress can also suppress hippocampal bcl-2 level. Therefore, we hypothesized that bcl-2 level would be reduced under stressful situations but increased by voluntary exercise. However, in this study, bcl-2 showed only a tendency of increase without statistical significance. A significant decrease in bcl-2 level has been observed under restraint stress in ovarian cells^3^. However, long-term swimming exercise does not change protein expression level of bcl-2^4^. In addition, bcl-2 level is increased by treadmill exercise^41^. Considering those reports, blc-2 expression levels do not always show the same results. Therefore, future research studies with various methodological approaches are needed to determine the role of blc-2 in stress. 

Caspase-3 is a protein that directly induces apoptosis^36^. An eight-week swimming exercise training has resulted in significantly reduced apoptosis and apoptosis marker expression in the hippocampus. In addition, swimming training has reduced casepase-3 protein expression^42^. As previously mentioned, exercise can result in effective suppression of apoptosis^43^. Kim et al.^21^ have reported that rat pups born from diabetic rats who performed treadmill exercise have shown suppression of apoptosis. This study limitation in studying the expression level of caspase-3 is that cleaved caspase-3 expression levels are usually presented in the results as its protein expression level instead of the full-length caspase-3. This study also showed that caspase-3 protein expression was suppressed by voluntary exercise in RSVE. On the contrary, it showed higher expression by restraint stress in RS. These results might be due to the fact that, after 1 h of restraint stress, rats freely participated the voluntary exercise. This might have affected caspase-3 protein expression. It has been reported that prolonged restraint stress load can result in the increase of caspase-3 and apoptosis in rats^44^. 

Cortisol, a typical stress hormone, is secreted from the adrenal cortex of the kidney. Cortisol can respond to stimuli such as stress. In this study, cortisol was increased significantly by restraint stress. However, it showed significantly lower level during voluntary exercise after restraint stress. Interestingly, RSVE had significantly lower cortisol levels than CON. It has been reported that cortisol level is significantly increased by restraint stress and the trend has started from the first day of restraint^5^. When Cynomolgus monkey is tied in a chair to limit its mobility, its cortisol concentration is increased^45^. Similarly, as a stress response of bottlenose dolphin, its cortisol level is continuously increased until the time it is discharged from the initial capture^46^. Serum cortisol concentration is also increased 5-fold compared to that in the control group after one hour of restraint^47^. 

The changes of serum cortisol concentration in Wistar rats under forced swim and exercise restraint situations have been studied previously^2^. It was found that serum cortisol concentrations were significantly increased under the restraint condition and the forced swimming exercise situation. Additional forced swimming exercise after restraint stress additionally increased the cortisol levels, although not statistically significant. These results suggested that restraint brings more stress than forced swimming exercise. In addition, it has been reported that cortisol level is significantly increased by resistance training^48^ or intermittent exercise training^49^. However, these researches were focused on relatively high intensity exercise that could induce exhaustion. Under long term stressful situations, a voluntary exercise does not have significant effect on cortisol level^50^. Therefore, under specific stress situations, participation of voluntary exercise may be one of the way to reduce stress. 

In summary, the results of the present study suggest that restraint stress for a long period of time is likely to accelerate aging and induce apoptosis. In addition, considering the changes of cortisol concentrations, long-term stress exposure can increase the prevalence of various metabolic disorders. An alternative way to reduce stress, voluntary exercise can bring positive effect on health because it inhibits apoptosis and suppress cortisol level. Further research studies are needed to determine the effect of both nutritional intake and exercises on health under a variety of stressful situations.
